# Supplementary material for: Characterization of a prototype rapid kilovoltage x‐ray image guidance system designed for a linear accelerator radiation therapy unit
Source: J Appl Clin Med Phys. 2025 Feb 26;26(5):e70060. doi: 10.1002/acm2.70060 (PMC12059271; doi:10.1002/acm2.70060)
Supplement: Supplementary file 1 — Supporting Information [file ACM2-26-e70060-s001.docx]

Characterization of a Prototype Rapid Kilovoltage X-ray Image Guidance System Designed for a Linear Accelerator Radiation Therapy Unit

**Theodore Arsenault MS^1,2^, Jonathan Arrue MS^1^, Kenneth Gregg MS^1^, Atallah Baydoun MD, PhD^1^, Atefeh Rezai MD^1^, Christian Langmack PhD^1^, Lauren Henke MD^1^, Daniel E Spratt MD^1^, Rojano Kashani PhD^1^, Alex Price PhD^1^**

^1^ Department of Radiation Oncology, University Hospitals Seidman Cancer Center, Cleveland, OH 44106

^2^ Department of Biomedical Engineering, Case Western Reserve University, Cleveland, Ohio 44106, USA

**Running Title:** “Characterization of a Prototype Rapid kV X-ray Guidance System for LINAC”

**Corresponding Author Information:**

Theodore Arsenault

*Theodore.Arsenault@UHhospitals.org*

University Hospitals Seidman Cancer Center, Bolwell B182

11100 Euclid Avenue, Cleveland, OH, 44116

**Abstract**

**Purpose:** This study evaluates a novel cone-beam computed tomography (CBCT) imaging solution integrated onto a conventional C-arm linear accelerator (linac) with increased gantry speed. The purpose is to assess the impact of improved imaging hardware and reconstruction algorithms on image quality.

**Methods:** Hypersight-CBCT (HS-CBCT) system was compared with the original system (OG-CBCT) on a TrueBeam linac. Performance tests included mechanical, geometric, setup accuracy, and image quality assessment. Image quality metrics were evaluated using conventional CBCT reconstruction and advanced iterative reconstruction (iCBCT), fast iCBCT, and iCBCT with metal artifact reduction. Dosimetry measurements were acquired.

**Results:** The HS-CBCT system acquired images in 24.0-44.0 seconds (half trajectory/full trajectory), faster than the OG-CBCT system's acquisition time of 33.5-60.5 seconds (half trajectory/full trajectory). The HS-CBCT system's faster gantry speed resulted in comparable image quality to the OG-CBCT. It improved high-contrast resolution, modulation transfer function, and low-contrast visibility. The faster gantry speed also produced lower radiation doses. The system's uniformity and resolution also improved, particularly with full-fan acquisition techniques.

**Conclusion:** The novel HS-CBCT system on a conventional C-arm linac exhibits superior imaging capabilities compared to the OG-CBCT. Faster gantry speed, larger imaging area, and advanced reconstruction algorithms contribute to enhanced image quality and reduced dose. The study provides comprehensive insights into the new system's performance, serving as a benchmark for future linac installations and highlighting potential benefits in clinical applications. Further investigations are suggested for 4D acquisitions and long-term machine performance.

# **1.** **Introduction**

Image-guided radiation therapy (IGRT) has become an important part of radiotherapy treatments in recent years, with most modern treatment devices equipped with an integrated on-board imaging system. One such imaging technique is on-board kilovoltage cone beam computed tomography (kV-CBCT). This technology provides high-resolution images of tumor and surrounding tissues to guide patient positioning and deliver the radiation treatment with precision. kV-CBCT has significantly improved radiation therapy accuracy, reducing margins, increasing therapeutic ratios, and resulting in fewer side effects for patients.[1–3].

Although on-board kV-CBCT has led to improvements in alignment accuracy, cone-beam-based geometry is more susceptible to imaging artifacts, which has limited kV-CBCT image utility in other aspects of radiotherapy workflows[4–6]. In this geometry, increased acquisition kV field-size has a higher proportion of scattered X-rays affecting image quality when compared to fan-beam CT geometries[4]. Additionally, the slower acquisition seen on conventional C-arm linacs at approximately 1 rotation per minute (RPM), increases the motion artifact likelihood within the kV-CBCT image. It has been shown that increased RPM leads to fewer motion artifacts, which leads to increases in-vivo image quality [6]. Detectors with improved detective quantum efficiency (DQE) and advances in image reconstruction algorithms have also led to improved image quality [7]. A similar imaging solution evaluated in this study has been introduced on an O-ring linac and demonstrated improved image quality compared to previous C-arm linac versions evaluated in this study. However, the O-ring imaging solution has a different imaging panel size, gantry rotation speed, and overall geometric configuration when compared to C-arm linacs[8], thus limiting a controlled comparison, which we aim to demonstrate here in this study. Additionally, this advanced imaging solution has yet to be evaluated in literature when installed a on a C-arm linac.

In the current study, we evaluated a novel CBCT imaging solution with higher DQE installed on a conventional C-arm linac capable of increased gantry speeds during cone-beam acquisition with a larger kV imaging area. A suite of image quality tests was performed on a previous version of the C-arm linac CBCT system and compared to the novel C-arm CBCT imaging system. We hypothesized that this C-arm linac imaging system would have improved image quality due to the improved image reconstruction algorithms, faster gantry speed, and improved DQE. Additionally, we will report results from this novel C-arm linac imaging system for readers to reference during the commissioning of subsequent installations.

# **2.** **Materials & Methods**

## **2.1 Overview of kV IGRT System**

The TrueBeam v4.0 C-arm linac (Varian Medical Systems, Palo Alto, CA), includes major changes from earlier versions of on-board kV-CBCT, including integration of a new kV imaging panel (Hypersight) with a maximum imaging area of 43x43cm^2^ (RTI4343iL) compared to the standard 40x30cm^2^ (RTI4030iL) panel currently in use. We will refer to the novel C-arm linac imaging system as HS-CBCT system and the prior version as the Original CBCT (OG-CBCT) system. This new panel’s imaging hardware was also upgraded with an improved scatter grid ratio of 15:1 and an amorphous silicon detector. Additionally, an upgraded gantry rotation power source enabled a gantry speed increase to 9.0deg/sec (1.5 RPM) for HS-CBCT systems compared to the earlier versions' 6.0deg/sec (1 RPM).

The acquisition geometry for "Head" and "Image Gently" protocols used a full-fan geometry and partial trajectory with a 25cm diameter field-of-view (FOV), while "Pelvis," "Thorax," and "Pelvis Large" protocols used a half-fan geometry and full trajectory with a 46cm diameter FOV. All CBCT modes could reconstruct images using the conventional Feldkamp-Davis-Kress (FDK) algorithm. The HS-CBCT system offered a nonlinear reconstruction algorithm called iterative CBCT (iCBCT) for several protocols. The iCBCT algorithm produces superior image quality with less noise, more uniformity, and higher contrast-to-noise ratio (CNR) than the standard FDK reconstruction[8]. The iCBCT reconstruction utilized a statistical reconstruction process combined with a scatter correction algorithm (Acuros CTS) to ensure the reconstructions minimize image artifacts, enhance image sharpness, and improve overall image quality [8–11]. This imaging solution also enabled fast iCBCT (faster reconstruction times) and CBCT metal artifact reduction (MAR) algorithms. Post-acquisition, all image reconstruction algorithms were applied to the raw data to ensure consistency in comparison across different reconstruction techniques. A comparison of kV specifications of the OG-CBCT and HS-CBCT system are shown below in Table 1.

**Table 1.** kV CBCT specifications for the OG-CBCT and HS-CBCT systems. The HS-CBCT values that differ from the OG-CBCT are shown within the parentheses.

| **Deployed CBCT Modes** | **Head** | **Image Gently** | **Thorax** | **Pelvis** | **Pelvis Large** |
| --- | --- | --- | --- | --- | --- |
| **Voltage (kVp)** | 100 | 80 | 125 | 125 | 140 |
| **Tube current (mA)** | 15 | 20 | 15 | 60 | 75 |
| **Pulse duration (ms)** | 20 | 10 | 20 | 25 | 25 |
| **Frame rate (fps)** | 15 (22) | 15 (22) | 15 (22) | 15 (22) | 15 (22) |
| **Scan arc (degrees)** | 200 | 200 | 360 | 360 | 360 |
| **Gantry rotation speed (degrees/second)** | 6 (9) | 6 (9) | 6 (9) | 6 (9) | 6 (9) |
| **Scan duration (seconds)** | 33 (22.2) | 33 (22.2) | 60 (40) | 60 (40) | 60 (40) |
| **Number of Projections** | 500 | 500 | 900 | 900 | 900 |
| **Exposure (mAs)** | 150 | 100 | 270 | 1080 | 1688 |
| **Fan type** | Full fan | Full fan | Half fan | Full fan | Half fan |
| **Slice thickness (mm)** | 2 | 2 | 2 | 2 | 2 |
| **Reconstruction methods** | FDK, iCBCT (FDK, iCBCT, MAR) | FDK, iCBCT (FDK) | FDK | FDK, iCBCT (FDK, iCBCT, MAR) | FDK, iCBCT (FDK, iCBCT, MAR) |

## **2.2 Overview of kV Imaging Performance Characterization**

Following the national guidelines and AAPM Task Group Reports 46, 104, and 142 [12–14]. A series of tests, shown in Table S1, were performed to characterize the performance of both CBCT systems.

## **2.3 Mechanical, Geometric, and Patient Setup Accuracy Tests**

HS-CBCT system's mechanical accuracy was tested using kV imager projection offset and field edge accuracy. Tests were performed with the system's internal machine performance check (MPC) and Isocenter Calibration (IsoCal) tests[15]. The MPC software automatically analyzes kV images of the MPC drum phantom. Imager projection offset measures the distance between the imager center and treatment isocenter projection which is crucial for IsoCal calibration accuracy and CBCT image quality. A low value is necessary for accurate matching and optimal image quality. kV imager projection offset, and field edge accuracy were tracked over a period of two months to detect any deviation in the systems’ mechanical accuracy.

## **2.4 kV Image Quality Tests**

### **2.4.1 Phantoms used for kV image Quality.**

Two physical phantoms were used to assess CBCT and kV planar imaging protocols, respectively: the Catphan 604(The Phantom Laboratory, Salem, NY) and Leeds TOR18 Phantom (Leeds Test Objects Ltd, North Yorkshire, UK). Catphan is widely used in CBCT QA-related studies and is a foundational phantom for evaluating linac CBCT imaging systems[16–19]. It was chosen as the vendor supplies it with the linac/CBCT systems under investigation. An image of the Catphan phantom taken with the OG-CBCT and HS-CBCT can be seen in Figure S1.

The Leeds phantom is a common phantom designed to provide an ongoing check of imaging performance by testing 2D spatial resolution, 2D low contrast detectability, and Modulation Transfer Function (MTF).

### **2.4.2 Image Quality Analysis using the Catphan Phantom**

*Strategies for Computed Tomography (CT) uniformity*: The image uniformity module of Catphan was made from a uniform material with a CT number within 2% of water's density. To evaluate CT system precision, mean values of CT number were measured from a circular 400 mm^2^ region of interest (ROI) at different locations within the scan field: Center, top, bottom, left, and right. Uniformity was calculated using equation 1:

$$Uniformity = \left| \overline{CT}_{ROI, Periphery}-\overline{CT}_{ROI, center} \right|$$

( 1)

where $\overline{CT}_{ROI, center}$ and $\overline{CT}_{ROI, periphery}$ are the mean pixel value of the ROI at the center and periphery respectively. A lower number in the resultant value from the equation demonstrates improved uniformity.

*Strategies for measuring high contrast resolution:* The modulation transfer function (MTF) is a fundamental measure of spatial resolution applied to images with sharp edges. For 3D CBCT measurement, we used the Catphan phantom for 3D imaging. Studies report that 50% of the maximum MTF (MTF50) is an ideal parameter for comparing the sharpness of imaging systems. Since the human eye is sensitive to >10% of the maximum MTF (MTF10), we reported both MTF50 and MTF 10 [7]

All measurements of MTF were calculated using RIT v6.10 (Radiological Imaging Technology, Inc, Colorado Springs, CO) and are reported in lp/cm.

*Strategies for evaluating noise:* The noise power spectrum (NPS) provides a measure of the noise variance as a function of spatial frequency [20]. The NPS is calculated using the Catphan uniformity module (CTP486) using equation 2 as defined by ICRU Report 87:

$$NPS= \frac{\Delta_{x}\Delta_{y}}{N_{x}N_{y}} \frac{1}{N}\sum_{i=1}^{N} \left| DFT_{2D}\left( {ROI}_{i}-\bar{{ROI}_{i}} \right) \right|^{2}$$

( 2)

where $N_{x}, N_{y}$ are the number of pixels in the x and y direction respectively, N is the number of ROIs evaluated, $\bar{ROI_{i}}$ is the average of the region of interest, ${ROI}_{i}\left( x,y \right)$ is the $i$-th image, and $\Delta_{x}{, \Delta}_{y}$are the pixel sizes in mm[20].

*Measurement of low contrast resolution:* The Catphan phantom provides a module with varying material inserts, which we used to measure low contrast visibility% (LCV) as a surrogate for low contrast resolution.

The measurement procedure took the mean HU value within a region of interest (ROI). The LCV was calculated using the following equation [21]. A lower number in the resultant LCV equation represents improved low-contrast visibility.

$$LCV = \frac{2.75\left( \sigma_{ROI,poly}+\sigma_{ROI,LDPE} \right)}{\overline{P_{ROI,poly}} -\overline{P_{ROI,LDPE}}}$$

( 3)

### **2.4.3 Image Quality Analysis using the Leeds Phantom**

To test the onboard 2D planar imaging (XI) system, we used the Leeds phantom to measure the MTF50, MTF10, and CNR, as described above. All analysis was performed using RIT v6.10.

## **2.5 Dosimetry Measurement**

The computed tomography dose index (CTDI) phantom, an Exradin A101 CTDI chamber, and a Standard Imaging Max 4000 Electrometer were used in this study for kV volumetric imaging dose measurement. CTDI was used for this evaluation due to the IEC requirements for linac imaging dosimetry[22]. Readings were taken in the phantom for each protocol at the center and four peripheral positions. Different phantoms were used for the protocols: a 16 cm head phantom for “Head” and “Image Gently”, and a 32 cm body phantom for the other three. Measurements were performed with the default kVp and mAs settings in clinical mode, as shown in Table 1. These measured values were compared to the value reported by the CTDI value measured using the OG-CBCT system.

# **3.** **Results**

## **3.1 Volumetric Image Acquisition and Reconstruction Time**

Five imaging datasets of the Catphan phantom were acquired using default vendor settings and reconstructed using FDK, iCBCT (when available), iCBCTfast and iCBCT MAR (when available). Across all protocols a >30% decrease in scan time can be seen as shown in Table 1.

## **3.2 Mechanical and Geometric Accuracy**


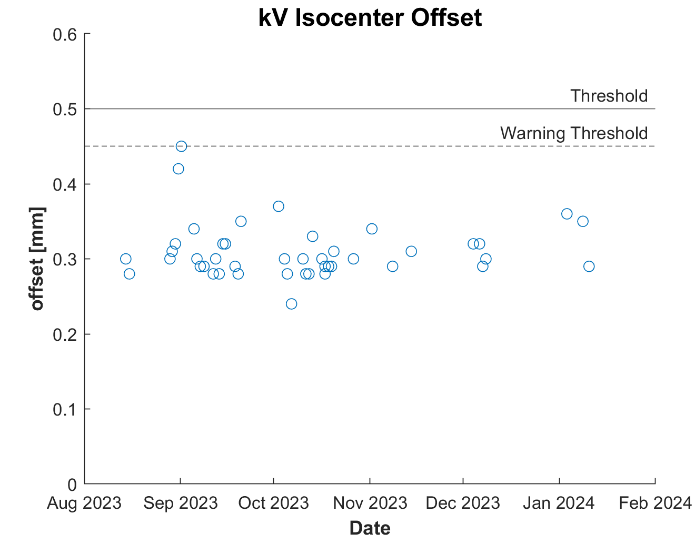

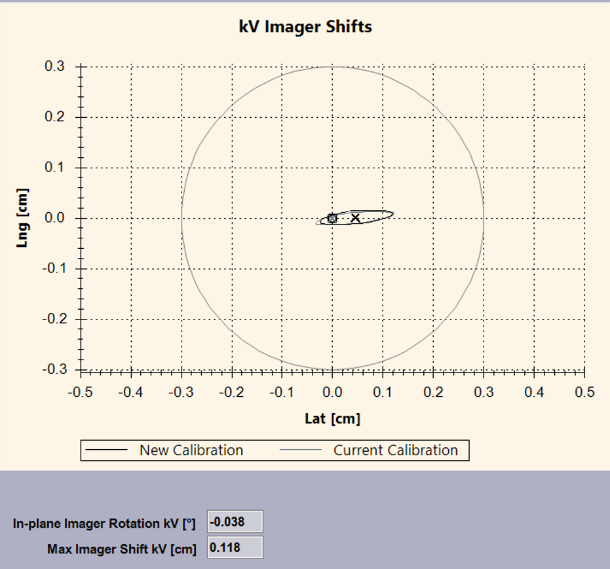


**Figure 1.** Varian TrueBeam tests provided by vendor. (a) MPC results for kV imager offset over a period of 6 months. (b)Varian IsoCal results from commissioning (thin line) and two months after acceptance (thick line).

**(a)**

**(b)**

Results from MPC and IsoCal tests reveal how the new kV imaging panel for the HS-CBCT system affects the kV imaging isocenter over time using the vendor-provided MPC phantom. Figure 1a illustrates the kV imager offset over 2 months. It revealed that the imager offset fell within the 0.5mm tolerance. The software flags all results that fall within 10% of the tolerance for MPC tests.

Figure 1b reveals the IsoCal results from acceptance (thin line) and 2 months after acceptance (thick line). As seen in Figure 1b, the two lines align well, denoting no meaningful change has occurred. The largest kV imager shift two months past acceptance was 1.18mm.

## **3.3 Image Quality Tests**

**3.3.1 Uniformity**


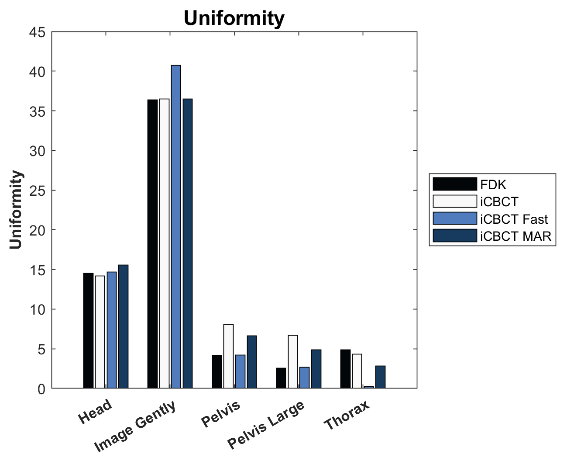

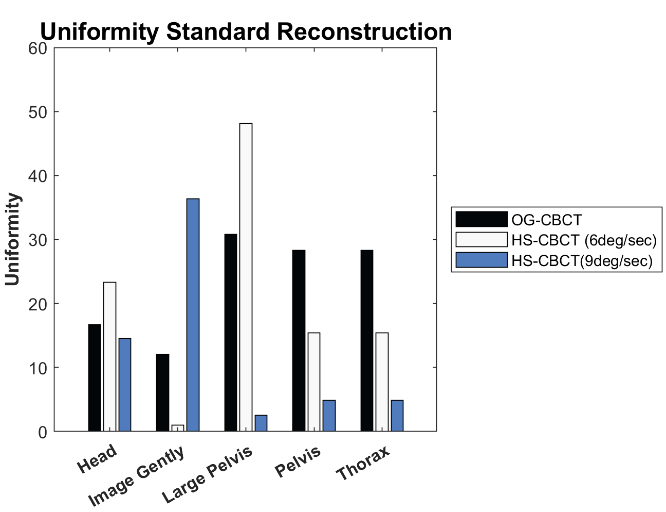

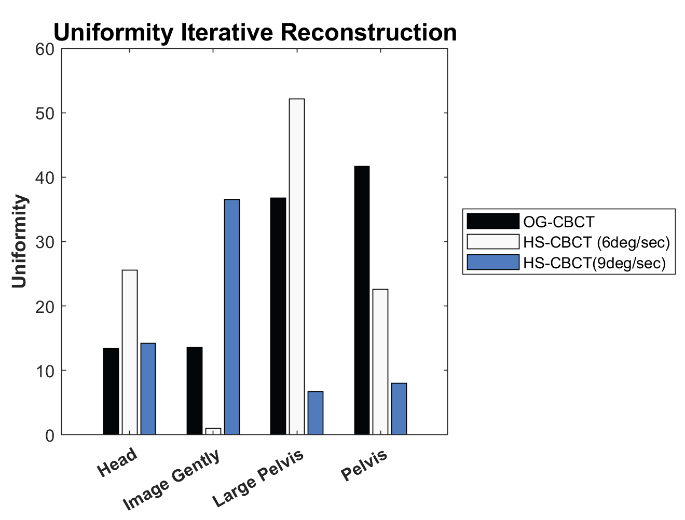


**Figure 2.** (a) Uniformity for five available scan protocols using four available reconstruction techniques on the HS-CBCT system. Uniformity of (b) standard reconstruction and (c) iCBCT for varying gantry speeds across various scan protocols.

**(a)**

**(b)**

**(c)**

Uniformity compared across 5 scan protocols is illustrated in Figure 2a. The largest variation in HU uniformity can be seen in the “Pelvis”, “Pelvis Large”, and “Thorax”. Uniformity was also compared when utilizing different gantry speeds during acquisition shown in figure 2b and 2c. The HS-CBCT system HS-CBCT (9 degrees/sec) tend to result in reduced uniformity across most anatomical sites, particularly for larger regions, while the slower scan speed (6 degrees/sec) produces more comparable uniformity to OG-CBCT.

**3.3.2 High Contrast Resolution Measurements**

**Table 2.** Comparison of 2D Contrast to Noise ratio and High contrast resolution (MTF in lp/cm) using the Leeds phantom for the OG-CBCT and HS-CBCT imaging systems.

|  |  | OG-CBCT System | | | HS-CBCT System | | |
| --- | --- | --- | --- | --- | --- | --- | --- |
| kV | **mAs** | **CNR** | **MTF50** | **MTF10** | **CNR** | **MTF50** | **MTF10** |
| 60 | 5.1 | - | - | - | 51.6 | 1.1 | 1.7 |
| 75 | 2.6 | 45.7 | 1.2 | 1.9 | 89.0 | 1.2 | 2.0 |
| 75 | 5.0 | 78.1 | 1.1 | 1.5 | 102.1 | 1.2 | 1.8 |
| 88 | 5.0 | 107.0 | 1.1 | 1.7 | 120.4 | 1.2 | 1.8 |
| 120 | 1.7 | 65.0 | 1.1 | 1.6 | 96.6 | 1.2 | 1.8 |
| 120 | 5.0 | 102.1 | 1.2 | 1.9 | 61.7 | 1.2 | 2.1 |

Table 2 compares 2D high contrast resolution using the Leeds phantom for OG-CBCT (RTI4030iL) and HS-CBCT (RTI4343iL) imaging systems. HS-CBCT reports a higher MTF10 than OG-CBCT for all tube techniques, and a greater CNR and MTF50 for 3 out of 5 techniques.

Table 3 presents the average noise power spectrum (NPS) and modulation transfer function (MTF) test results of OG- and HS-CBCT systems with two rotation speeds. For all tested protocols, HS-CBCT performed similarly or outperformed its predecessor, with the greatest improvements occurring in the “Head” and “Image Gently” protocols. In most cases, the HS-CBCT 6deg/sec scans yielded the highest resolution as shown in Table 3. Despite a slight decrease in spatial resolution, the 9deg/sec scans were comparable to the previous panel's spatial resolution with standard gantry rotation speed.

**Table 3.** Comparison of the average NPS, MTF50, and MTF10 (MTF in lp/cm) using the Catphan phantom for the OG-CBCT and HS-CBCT imaging systems.

| **Scan Information** | | **Average NPS** | | | **MTF50** | | | **MTF10** | | |
| --- | --- | --- | --- | --- | --- | --- | --- | --- | --- | --- |
| **Protocol** | **Recon** | **OG-CBCT** | **HS-CBCT 6deg/sec** | **HS-CBCT 9deg/sec** | **OG-CBCT** | **HS-CBCT 6deg/sec** | **HS-CBCT 9deg/sec** | **OG-CBCT** | **HS-CBCT 6deg/sec** | **HS-CBCT 9deg/sec** |
| Pelvis | FDK | 0.8 | 2.9 | 0.8 | 3.5 | 3.6 | 3.6 | 5.0 | 5.3 | 5.1 |
| Pelvis | iCBCT | 0.5 | 1.2 | 0.2 | 3.6 | 3.6 | 3.5 | 4.9 | 5.1 | 4.9 |
| Pelvis | iCBCTfast | - | 0.9 | 0.6 | - | 3.7 | 3.5 | - | 5.2 | 4.9 |
| Pelvis | iCBCT MAR | - | 2.1 | 0.5 | - | 3.7 | 3.5 | - | 5.16 | 4.9 |
| Head | FDK | 4.5 | 2.9 | 14.0 | 2.8 | 4.6 | 4.5 | 6.1 | 6.9 | 6.7 |
| Head | iCBCT | 7.1 | 1.2 | 3.3 | 2.9 | 4.7 | 4.6 | 5.3 | 6.3 | 6.1 |
| Head | iCBCTfast | - | 0.9 | 1.0 | - | 4.8 | 4.6 | - | 6.4 | 6.2 |
| Head | iCBCT MAR | - | 2.1 | 0.4 | - | 4.7 | 4.6 | - | 6.4 | 6.2 |
| Pelvis Large | FDK | 0.5 | 0.3 | 0.1 | 3.5 | 3.5 | 3.6 | 5.0 | 5.2 | 5.1 |
| Pelvis Large | iCBCT | 0.6 | 0.5 | 0.1 | 3.6 | 3.6 | 3.5 | 4.9 | 5.1 | 5.0 |
| Pelvis Large | iCBCTfast | - | 0.5 | 0.1 | - | 3.7 | 3.6 | - | 5.2 | 5.1 |
| Pelvis Large | iCBCT MAR | - | 0.5 | 0.4 | - | 3.6 | 3.6 | - | 5.1 | 5.0 |
| Thorax | FDK | 1.4 | 0.5 | 2.2 | 3.5 | 3.6 | 3.5 | 5.0 | 5.3 | 5.1 |
| Thorax | iCBCT | - | 0.3 | 0.8 | - | 3.6 | 3.5 | - | 5.1 | 5.0 |
| Thorax | iCBCTfast | - | 0.9 | 2.2 | - | 3.7 | 3.5 | - | 5.2 | 5.0 |
| Thorax | iCBCT MAR | - | 1.2 | 0.7 | - | 3.7 | 3.5 | - | 5.2 | 4.9 |
| Image Gently | FDK | 14.4 | 1.7 | 0.6 | 2.7 | 4.5 | 4.8 | 5.4 | 7.3 | 6.4 |
| Image Gently | iCBCT | 15.7 | 1.3 | 0.2 | 2.9 | 4.5 | 4.8 | 5.5 | 6.7 | 6.4 |
| Image Gently | iCBCTfast | - | 0.9 | 0.2 | - | 4.6 | 4.7 | - | 7.0 | 7.1 |
| Image Gently | iCBCT MAR | - | 2.4 | 0.1 | - | 4.1 | 4.8 | - | 6.4 | 6.4 |

Scanning protocols with gantry speed of 9 degrees/sec had a spatial resolution greater than or equal to 3.5 lp/cm for MTF50 and greater than 5 lp/cm for MTF10. iCBCT fast technique outperformed other iCBCT reconstructions.

### **3.3.3 Low Contrast**


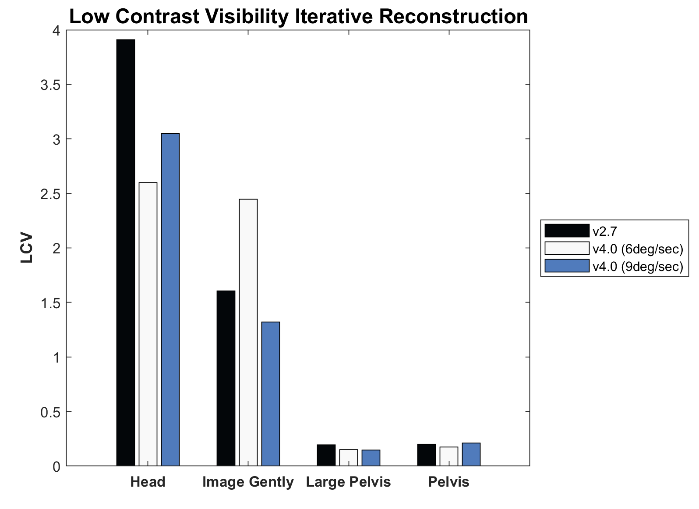

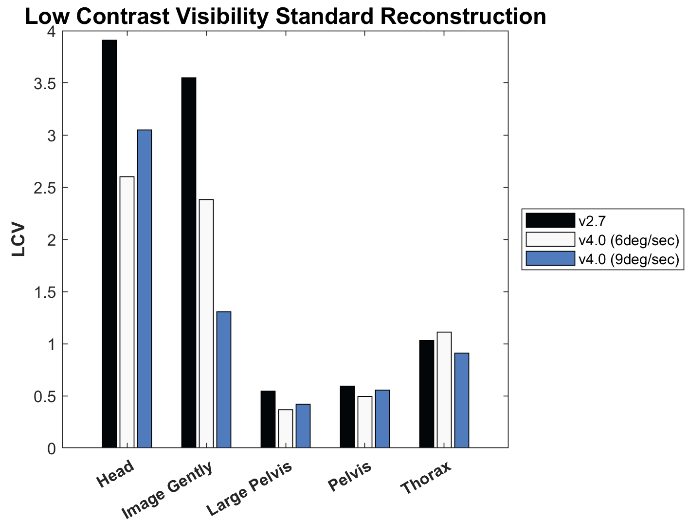

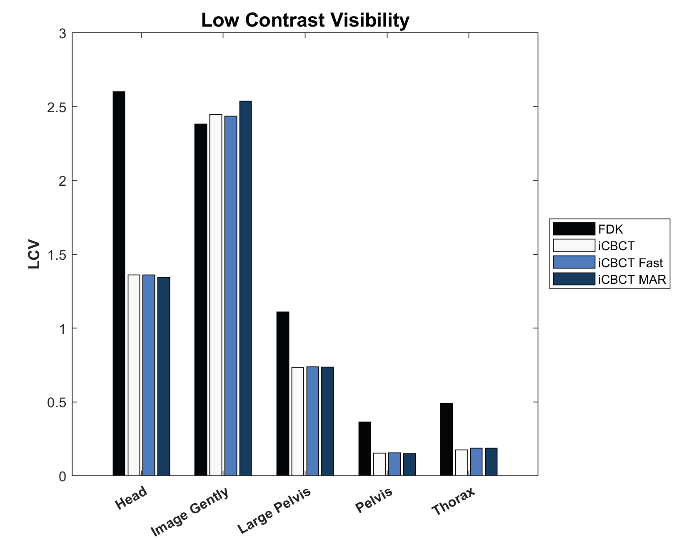


**(a)**

**Figure 3.** (a) Low contrast visibility for various scan protocols using 4 available reconstruction techniques. Low contrast visibility of (b) standard reconstruction and (c) iCBCT for varying gantry speeds across for various scan protocols

**(b)**

**(c)**


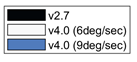

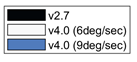

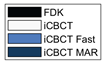


Low contrast resolution of the HS-CBCT system performed similarly compared to the prior panel as illustrated in Figure 3b and 3c. The low contrast visibility differences with the OG-CBCT lessened when using pelvic or thoracic protocols.

When comparing different reconstruction techniques, we saw that the FDK reconstruction technique generally had a worse low contrast visibility except for the “Image Gently” protocol. This is illustrated in Figure 3a and comparing Figures 3b and 3c.

## **3.4 Radiation Dose Measurement**


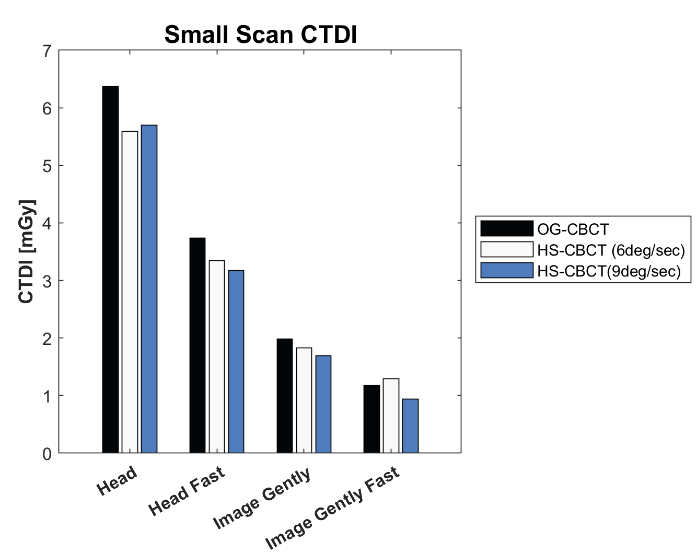

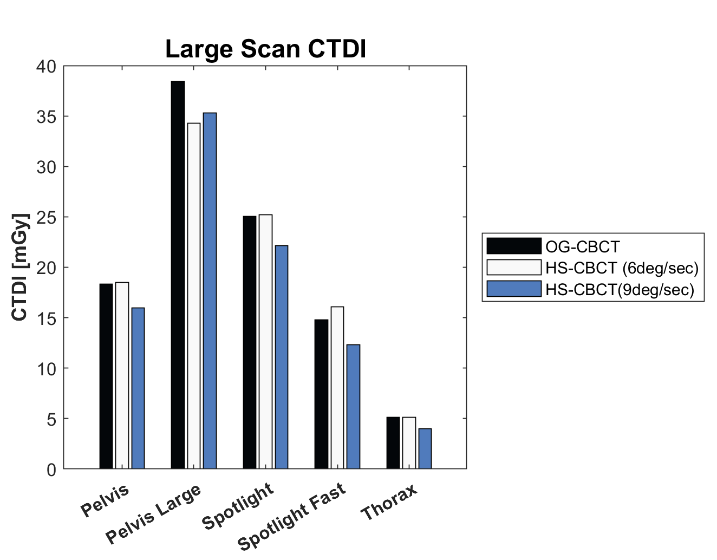


**Figure 4**. Intra-machine CTDI measurements using vendor supplied kV CBCT protocols. a) CTDI measurements taken with the 32cm phantom. b) CTDI measurements taken with the 16cm CTDI phantom.

**(b)**

**(a)**

Figure 4 shows intra-machine dose comparison from the OG-CBCT system and the two scan speeds available for HS-CBCT systems. Comparable results were observed across all machines, with version 4.0 measurements having a slightly lower CTDI with 9deg/sec gantry rotation. The “Large” protocol yielded higher CTDI values due to higher mAs needed. We also observed that the new panel yields a lower CTDI, with an average of –0.4 (-4.1 to 1.3) mGy and –1.5 (-3.1 to -0.2) mGy for 6deg/sec gantry rotation and 9deg/sec gantry rotation, respectively.

# **4.** **Discussion**

This study aimed to provide a comprehensive evaluation of a new onboard imaging system performance that offered a 43x43cm^2^ imaging area and could acquire kV CBCT scans at a gantry speed of 9deg/sec. We conducted a comparative analysis of HS-CBCT performance relative to a previous CBCT system. We used an AAPM guidelines-based approach to ensure the reproducibility of our findings.

The HS-CBCT system in this investigation demonstrated that increased gantry speed capability led to decreases in image acquisition time. However, iterative reconstruction techniques have been shown to result in increased reconstruction times [23] nullify time gains made with increased gantry speed. Despite this, in clinical practice, such as for a breath-hold treatment, obtaining on-board images in as little as 1-2 breath-holds will improve image quality needed for patient alignment [24]. This may improve confidence during image registration, leading to faster image registration times. Fewer breath holds can also improve patient comfort/compliance during breath-hold treatments [25]. Additionally, enhanced gantry speed did not impact geometric accuracy and reproducibility of the kV mechanical components, making the entire system mechanically sound during our investigation. When comparing to previous studies of the geometric accuracy of the kV system, the HS-CBCT performed similarly to OG-CBCT systems with smaller panels [26,27].

We found variations in our uniformity and analysis in among gantry speed, kVp, fan-geometry, and panel type. A higher kVp protocol had a substantial impact on uniformity in the "Large Pelvis" protocol which can be seen in other studies [28,29]. The significant increase in uniformity may be due to higher kVp in conjunction with a half-fan beam and full trajectory. Uniformity is multi-factorial function of imager type, kVp and mAs. This was seen in the work by Kim et al where there was variability across types[30].  However, increased gantry speed mitigated the worse uniformity seen with the "Large Pelvis" protocol. Increased gantry speed improved uniformity across all half-fan image acquisition techniques. Improved uniformity with faster gantry rotation and in-vivo reduction in motion artifact due to faster gantry speed should lead to overall improvements in image quality [24]. However, full-fan techniques showed more variation in uniformity results across panel type and gantry speed. Cai et. al. also saw varied uniformity with "Head" type protocols in their analysis in a separate O-ring linac CBCT system manufactured by the same vendor [7]. Additionally, there was no trend of image reconstruction choice on uniformity. Previous studies found that uniformity might improve somewhat, although not significantly [28,30,31]. The increased gantry speed may have an effect on the panel through sampling rate of the detector as the same amount of projections are acquired in a short amount of time, but further analysis needs to be done.

Variations in average NPS were found across all protocols. The “Image Gently” protocol showed the most significant decrease in average NPS between OG-CBCT and HS-CBCT. Scanning at 9deg/sec resulted in an overall decrease in average NPS, except for the “Thorax” and “Head” protocol. Optimizing scan protocols and reconstruction methods tailored to specific anatomical regions can significantly improve image quality.

The HS-CBCT panel improved the resolution of kV planar acquisition, which should enhance HS-CBCT resolution [32]. Full-fan acquisition techniques showed a significant improvement in resolution with HS-CBCT. However, the improvement was not as substantial with half-fan acquisition techniques (Table 2). Faster gantry rotation speeds led to slightly lower resolution metrics but should result in fewer motion artifacts.

Panel type appeared to positively impact LCV in almost all scenarios except for the “Image Gently” protocol with an iCBCT reconstruction, most likely due to the lower kVp value. When comparing panel types, we also demonstrated that full-fan techniques were much more varied in LCV. Additionally, the iCBCT-based reconstruction had a more consistent improvement in LCV in the HS-CBCT system compared to the FDK algorithm. This is like other findings where iCBCT has demonstrated improvements in LCV [32]. Gantry speed did not substantially impact LCV in contrast to uniformity. When looking at other investigations of CBCTs with faster gantry rotation speeds than 6deg/sec, we also see minimal differences in LCV[7]. Although not tested, adjusting the filter and/or other imaging reconstruction parameters may lead to further improvements in LCV.

When evaluating dose, due to the improved DQE of the HS-CBCT system, less dose is needed to obtain similar image quality [33]. We largely observed this effect but demonstrated that faster gantry rotation speeds had a greater impact on reducing dose during image acquisition. This has also been demonstrated in previous investigations into CBCT systems [7,34]. While the kVp and mAs are the same between 6deg/sec and 9deg/sec, an analysis of the projection metadata and DICOM metadata revealed that exposure time per projection was slightly lower for the 9deg/sec scan which yields a lower overall dose.

It is worth noting that O-ring systems use similar imaging systems with faster rotation and image acquisition times. Comparing with C-arm linac design could reveal insights into the scalability and adaptability of such technologies in different clinical settings. Future investigations could explore how different gantry designs affect system performance and clinical outcomes.

Our study can serve as a benchmark for clinics commissioning the HS-CBCT system. We followed AAPM guidelines and compared HS-CBCT to the previous version, testing its properties, image quality, and impact on workflow.

Despite its numerous advantages, the HS-CBCT system still has some limitations. The rapid gantry speed has yet to be thoroughly evaluated in the settings of 4D acquisitions, which is crucial when dealing with respiratory motion, and we do not understand how increased gantry speed impacts machine performance over time. Increased gantry speed and its impacts could manifest in imaging/machine isocenter stability due to increased demands on the gantry motors. Additionally, we did not comprehensively evaluate HU accuracy in this investigation. This is due to expected subsequent algorithm updates on the HS-CBCT system, which will impact HU measurements. In a forthcoming article our group performed a follow-up exhaustive investigation, outside the scope of this work, Involving HU characterization and dose calculation on the HU CBCT[35].

The performance of the new RTI4343iL kV panel on TrueBeam v4.0 or higher was evaluated by multiple image quality phantoms following the guidelines of the AAPM and vendor recommendations. Overall, the system met or exceeded recommendations.

**Credit Author Contributions:**

**Theodore Arsenault MS:** Conceptualization, Data Curation, Investigation, Writing- Original Draft **Jonathan Arrue MS & Kenneth Gregg MS:** Data curation, Writing –Review and Editing**, Atallah Baydoun MD, Atefeh Rezai MD, Christian Langmack PhD, Lauren Henke MD, Daniel Spratt MD, & Rojano Kashani PhD:** Writing – Review and Editing**, Alex Price PhD:** Supervision, Writing-Review and Editing

# **Conflict of Interest**

The authors declare the following financial interests/personal relationships which may be considered as potential competing interests:

- LusoPalex: honoraria/speaker/travel
- ViewRay Systems, Inc (no current, but all within the past one year): medical advisory board, CNS and GI advisory committees, honoraria/travel
- Varian Medical Systems/Siemens Healthineers:
  - Consulting fees, trial design, unrelated to this project
  - research grant funding, provided to my institution, related to this project
  - speaker/honoraria, unrelated to this project

**Data Availability Statement**

The data that support the findings of this study are available from the corresponding author upon reasonable request

# **Declaration of Generative AI and AI-assisted technologies in the writing process**

Statement: During the preparation of this work the author(s) used Grammarly in order to decrease word count and revise language. After using this tool/service, the author(s) reviewed and edited the content as needed and take(s) full responsibility for the content of the publication.

# **6.** **References**

[1] Nabavizadeh N, Elliott DA, Chen Y, Kusano AS, Mitin T, Thomas CR, et al. Image Guided Radiation Therapy (IGRT) practice patterns and IGRT’s impact on workflow and treatment planning: Results from a national survey of american society for radiation oncology members. Int J Radiat Oncol Biol Phys 2016;94:850–7. https://doi.org/10.1016/j.ijrobp.2015.09.035.

[2] Martins L, Couto JG, Barbosa B. Use of planar kV vs. CBCT in evaluation of setup errors in oesophagus carcinoma radiotherapy. Reports of Practical Oncology and Radiotherapy 2016;21:57–62. https://doi.org/10.1016/j.rpor.2015.10.005.

[3] Den RB, Doemer A, Kubicek G, Bednarz G, Galvin JM, Keane WM, et al. Daily Image Guidance With Cone-Beam Computed Tomography for Head-and-Neck Cancer Intensity-Modulated Radiotherapy: A Prospective Study. Int J Radiat Oncol Biol Phys 2010;76:1353–9. https://doi.org/10.1016/j.ijrobp.2009.03.059.

[4] Lechuga L, Weidlich GA. Cone Beam CT vs. Fan Beam CT: A Comparison of Image Quality and Dose Delivered Between Two Differing CT Imaging Modalities. Cureus 2016;8. https://doi.org/10.7759/cureus.778.

[5] Bissonnette JP, Balter PA, Dong L, Langen KM, Lovelock DM, Miften M, et al. Quality assurance for image-guided radiation therapy utilizing CT-based technologies: A report of the AAPM TG-179. Med Phys 2012;39:1946–63. https://doi.org/10.1118/1.3690466.

[6] Srnivasan K, Mohammadi M, Shepherd J. Applications of linac-mounted kilovoltage Cone-beam Computed Tomography in modern radiation therapy: A review. Pol J Radiol 2014;79:181–93. https://doi.org/10.12659/PJR.890745.

[7] Cai B, Laugeman E, Mazur TR, Park JC, Henke LE, Kim H, et al. Characterization of a prototype rapid kilovoltage x-ray image guidance system designed for a ring shape radiation therapy unit. Med Phys 2019;46:1355–70. https://doi.org/10.1002/mp.13396.

[8] Hu Y, Arnesen M, Aland T. Characterization of an advanced cone beam CT (CBCT) reconstruction algorithm used for dose calculation on Varian Halcyon linear accelerators. Biomed Phys Eng Express 2022;8. https://doi.org/10.1088/2057-1976/ac536b.

[9] Wang A, Maslowski A, Messmer P, Lehmann M, Strzelecki A, Yu E, et al. Acuros CTS: A fast, linear Boltzmann transport equation solver for computed tomography scatter – Part II: System modeling, scatter correction, and optimization. Med Phys 2018;45:1914–25. https://doi.org/10.1002/mp.12849.

[10] Peterlik I, Strzelecki A, Lehmann M, Messmer P, Munro P, Paysan P, et al. Reducing residual-motion artifacts in iterative 3D CBCT reconstruction in image-guided radiation therapy. Med Phys 2021;48:6497–507. https://doi.org/10.1002/mp.15236.

[11] Fessler JA. Penalized Weighted Least-Squares Image Reconstruction for Positron Emission Tomography. vol. 13. 1994.

[12] Yin F-F, Wong J. TG 104 :The Role of In-Room kV X-Ray Imaging for Patient Setup and Target Localization. vol. 40. 2009.

[13] Klein EE, Hanley J, Bayouth J, Yin FF, Simon W, Dresser S, et al. Task group 142 report: Quality assurance of medical acceleratorsa. Med Phys 2009;36:4197–212. https://doi.org/10.1118/1.3190392.

[14] Kutcher GJ, Coia L, Gillin M, Hanson WF, Leibel S, Morton RJ, et al. AAPM’s TG-46 Comprehensive QA for radiation oncology. Med Phys 1994;21:581–618.

[15] Barnes MP, Greer PB. Evaluation of the truebeam machine performance check (MPC) geometric checks for daily IGRT geometric accuracy quality assurance. J Appl Clin Med Phys 2017;18:200–6. https://doi.org/10.1002/acm2.12064.

[16] Andersen AG, Park YK, Elstrøm UV, Petersen JBB, Sharp GC, Winey B, et al. Evaluation of an a priori scatter correction algorithm for cone-beam computed tomography based range and dose calculations in proton therapy. Phys Imaging Radiat Oncol 2020;16:89–94. https://doi.org/10.1016/j.phro.2020.09.014.

[17] Stanley DN, Papanikolaou N, Gutiérreza AN. An evaluation of the stability of image-quality parameters of Varian on-board imaging (OBI) and EPID imaging systems. J Appl Clin Med Phys 2015;16:87–98. https://doi.org/10.1120/jacmp.v16i2.5088.

[18] Li Y, Jiang Y, Liu H, Yu X, Chen S, Ma D, et al. A phantom study comparing low-dose CT physical image quality from five different CT scanners. Quant Imaging Med Surg 2022;12:766–80. https://doi.org/10.21037/qims-21-245.

[19] Husby E, Svendsen ED, Andersen HK, Martinsen ACT. 100 days with scans of the same Catphan phantom on the same CT scanner. J Appl Clin Med Phys 2017;18:224–31. https://doi.org/10.1002/acm2.12186.

[20] Bentzen SM, Boone JM, Burns DT, Dawson P, Fantuzzi E, Gahbauer RA, et al. Journal of the ICRU 2013.

[21] Stock M, Pasler M, Birkfellner W, Homolka P. UKPMC Funders Group Image quality and stability of image-guided radiotherapy ( IGRT ) devices : A comparative study. Measurement 2010;93:1–7. https://doi.org/10.1016/j.radonc.2009.07.012.Image.

[22] International Electrotechnical Commission. Medical electrical equipment - Part 2-44: Particular requirements for the basic safety and essential performance of X-ray equipment for computed tomography. 2009.

[23] Vaniqui ana, R SchynS loTTe J, almeida iSaBel, Van deR heyden Br, podeSTa maRk, VeRhaeGen fRank, et al. The effect of different image reconstruction techniques on pre-clinical quantitative imaging and dual-energy CT. 2019.

[24] Robar JL, Cherpak A, MacDonald RL, Yashayaeva A, McAloney D, McMaster N, et al. Novel Technology Allowing Cone Beam Computed Tomography in 6 Seconds: A Patient Study of Comparative Image Quality. Pract Radiat Oncol 2023. https://doi.org/10.1016/j.prro.2023.10.014.

[25] Reitz D, Walter F, Schönecker S, Freislederer P, Pazos M, Niyazi M, et al. Stability and reproducibility of 6013 deep inspiration breath-holds in left-sided breast cancer. Radiation Oncology 2020;15. https://doi.org/10.1186/s13014-020-01572-w.

[26] Pearson M, Eaton D, Greener T. Long-term experience of MPC across multiple TrueBeam linacs: MPC concordance with conventional QC and sensitivity to real-world faults. J Appl Clin Med Phys 2020;21:224–35. https://doi.org/10.1002/acm2.12950.

[27] Barnes MP, Pomare D, Menk FW, Moraro B, Greer PB. Evaluation of the truebeam machine performance check (MPC): OBI X-ray tube alignment procedure. J Appl Clin Med Phys 2018;19:68–78. https://doi.org/10.1002/acm2.12445.

[28] Mao W, Gardner SJ, Snyder KC, Wen NW, Zhong H, Li H, et al. On the improvement of CBCT image quality for soft tissue-based SRS localization. J Appl Clin Med Phys 2018;19:177–84. https://doi.org/10.1002/acm2.12470.

[29] Yang B, Geng H, Chang TYA, Tse MY, Lam WW, Huang CY, et al. Clinical implementation of kVCT-guided tomotherapy with ClearRT. Phys Eng Sci Med 2022;45:915–24. https://doi.org/10.1007/s13246-022-01162-y.

[30] Kim E, Park YK, Zhao T, Laugeman E, Zhao XN, Hao Y, et al. Image quality characterization of an ultra-high-speed kilovoltage cone-beam computed tomography imaging system on an O-ring linear accelerator. J Appl Clin Med Phys 2024;25. https://doi.org/10.1002/acm2.14337.

[31] Lim R, Penoncello GP, Hobbis D, Harrington DP, Rong Y. Technical note: Characterization of novel iterative reconstructed cone beam CT images for dose tracking and adaptive radiotherapy on L-shape linacs. Med Phys 2022;49:7715–32. https://doi.org/https://doi.org/10.1002/mp.15943.

[32] Taneja S, Barbee DL, Rea AJ, Malin M. CBCT image quality QA: Establishing a quantitative program. J Appl Clin Med Phys 2020;21:215–25. https://doi.org/10.1002/acm2.13062.

[33] Singh V, Jain A, Bednarek DR, Rudin S. Limitations of anti-scatter grids when used with high resolution image detectors. Medical Imaging 2014: Physics of Medical Imaging 2014;9033:903362. https://doi.org/10.1117/12.2043063.

[34] Santoso AP, Song KH, Qin Y, Gardner SJ, Liu C, Chetty IJ, et al. Evaluation of gantry speed on image quality and imaging dose for 4D cone-beam CT acquisition. Radiation Oncology 2016;11. https://doi.org/10.1186/s13014-016-0677-8.

[35] Gregg K, Arsenault T, Rezaei A, Kashani R, et al. Hounsfield Unit characterization and dose calculation on a c-arm linac with novel on-board cone-beam computed tomography feature and advanced reconstruction algorithms. (Forthcoming).

# Supplementary Material

**Table S1.** kV imager tests performed with the Phantom used for each test.

| Test Detail |  | Phantom |
| --- | --- | --- |
| Geometric/Mechanical Accuracy | kV imager shift/rotation  kV imager offset projection  MV/kV coordinate coincidence | MPC Drum Phantom,  isoCube phantom |
| Image Quality | Spatial Resolution  Low-contrast resolution  CT number Uniformity and Noise  CT number Accuracy  Slice Thickness  Spatial Integrity | Catphan 604, LeedsTOR18 |
| kV x-ray Tube | kVp, mAs, ms, HVL | Cobia |
| Dosimetry | kV CBCT dose accuracy  kV CBCT dose consistency | CTDI phantom |


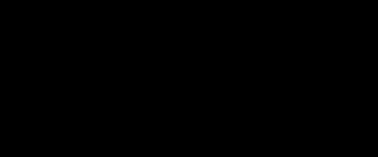

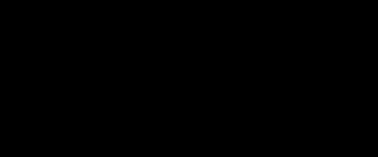

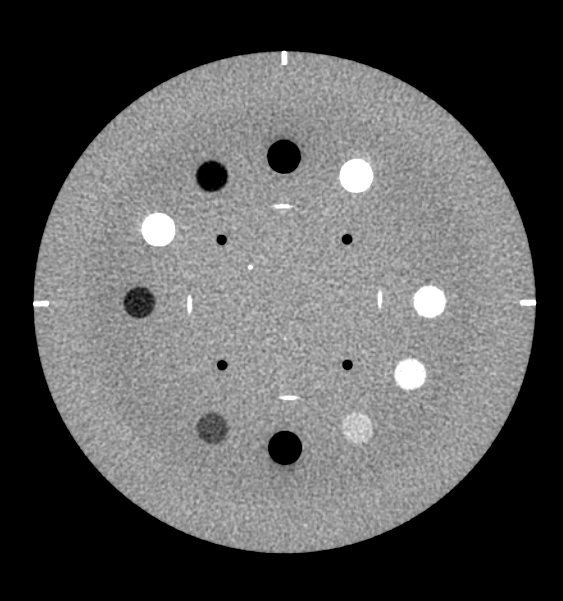

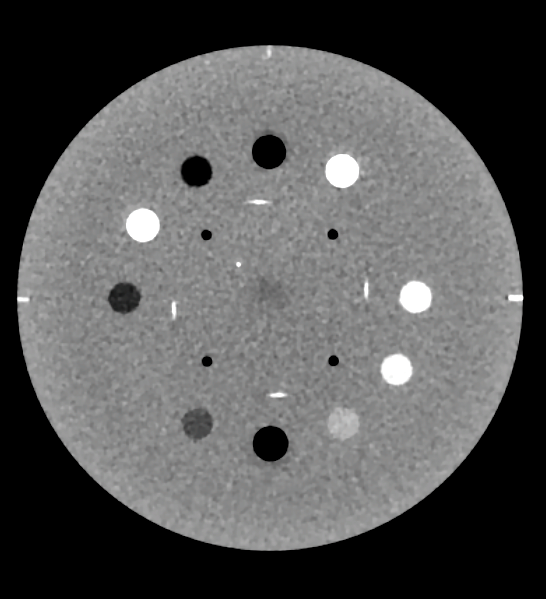

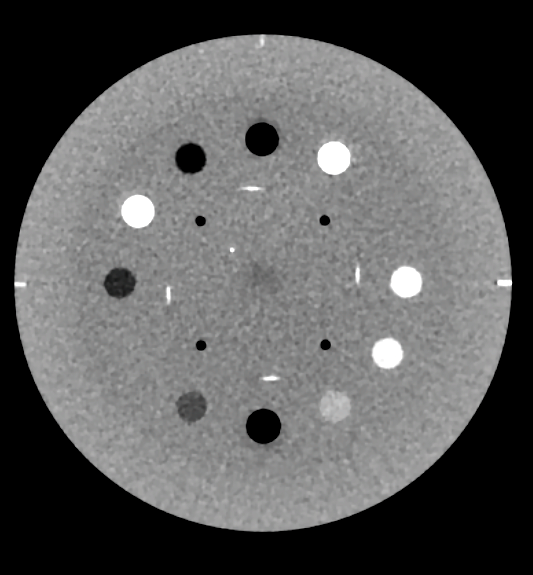

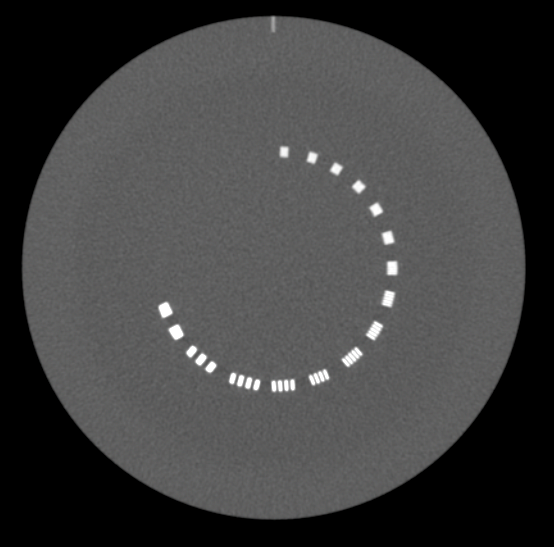

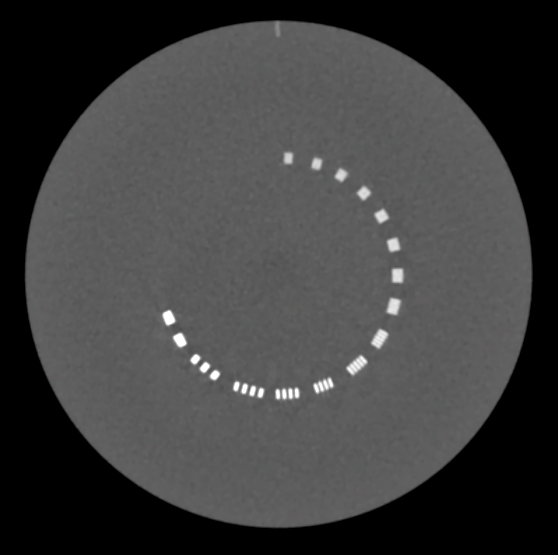

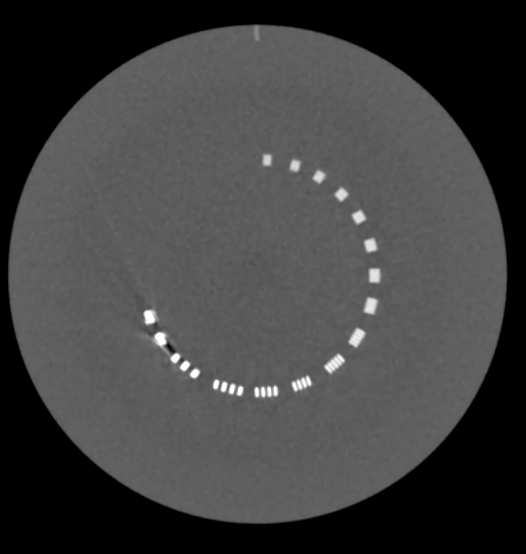


**(a)**

**(b)**

**(c)**

**(d)**

**(e)**

**(f)**

**Figure S1.** Comparison of CATPHAN using the head protocol. The top row depicts the high contrast resolution module for a) OG-CBCT with FDK reconstruction, b) HS-CBCT reconstruction, and c) HS-CBCT iCBCT reconstruction. The bottom row depicts the materials module for d) OG-CBCT with FDK reconstruction, e) HS-CBCT reconstruction, and f) HS-CBCT iCBCT reconstruction
